# Efficacy of radiotherapy and radiotherapy with hyperthermia to delay change of systemic therapy in patients with metastatic melanoma

**DOI:** 10.1016/j.ctro.2025.101024

**Published:** 2025-08-05

**Authors:** Paulina Chmiel, Mateusz Jacek Spałek, Hanna Koseła-Paterczyk, Piotr Łukasz Rutkowski, Paulina Jagodzińska-Mucha, Paweł Rogala, Katarzyna Kozak, Maria Telejko, Aneta Maria Borkowska

**Affiliations:** a Department of Soft Tissue/Bone Sarcoma and Melanoma, Maria Sklodowska-Curie National Research Institute of Oncology, Warsaw, Poland; b Department of Radiotherapy I, Maria Sklodowska-Curie National Research Institute of Oncology, Warsaw, Poland

**Keywords:** Melanoma, Immune checkpoint inhibitors, ICIs, BRAF/MEK, radiotherapy, RT, Hyperthermia, HT

## Abstract

**Background:**

The use of radiotherapy (RT) and RT with hyperthermia (HT) as a local treatment for oligoprogression in many tumors is becoming increasingly prevalent. However, there is currently limited data regarding RT efficacy in prolonging systemic therapy in melanoma. To address this lack of evidence, we conducted a single-institution study to establish the role of RT and RT + HT in delaying the time to systemic treatment switch in metastatic melanoma (MM).

**Methods:**

Patients with MM who received RT or RT + HT for oligoprogressive lesions at the referral center between 2018 and 2023 were identified. Oligoprogression was defined as up to five progressive metastases. Systemic treatment included immunotherapy and BRAF/MEK inhibitors. All patients who received radiotherapy to the brain and lungs were excluded. The primary endpoint was time to the next systemic therapy (TTNST) after RT/RT + HT. The secondary endpoints included overall survival (OS) and progression-free survival (PFS). Factors influencing TTNST were analyzed.

**Results:**

156 patients were included, 82 patients had RT + HT while 74 had RT only. The median follow-up was 23 months (15.2–34). Immunotherapy was used in 82.7 % and BRAF/MEK inhibitors in 17.3 % of patients. The median TTNST for the overall cohort was 26 months (95 % CI: 14.5- NA), the mTTNST for patients treated with RT + HT was 14 months (95 % CI: 11.4-NA), and for patients treated with RT as sole treatment, it was 28 months (95 % CI: 18.3-NA) (p = 0.6). The median PFS from local treatment was 8 months (95 % CI: 5.1–16) for patients who received RT and 10 months (95 % CI: 5.6–12.6) for patients who received RT + HT (p = 0.9). The median OS from RT was 50 months (95 % CI: 40- NA) for patients who received RT and was not reached for patients who received RT + HT (p = 0.031). None of the analyzed factors influenced median TTNST.

**Conclusion:**

Both RT and RT + HT have been demonstrated to extend the duration of systemic treatment in patients with metastatic melanoma. The combination of RT + HT is suggested to be more efficacious than RT alone in the therapy of oligoprogressive disease during systemic treatment of patients, especially for OS.

## Highlights

• RT and RT + HT delayed the need to change systemic therapy in metastatic melanoma.
• RT + HT improved OS compared to RT alone, despite a shorter median TTNST.
• No clinical or treatment-related factors were associated with longer TTNST.
• Both RT and RT + HT are suitable options for pts ineligible for further therapies.

## Introduction

The advent of immune checkpoint inhibitors (ICIs) and targeted therapy with BRAF/MEK inhibitors (BRAFi/MEKi) has profoundly transformed the landscape of melanoma treatment, leading to marked improvements in outcomes for patients with metastatic melanoma (MM) [[1], [2], [3]]. Current guidelines advocate for the utilization of PD-1 blockade (nivolumab, pembrolizumab), PD-1 blockade in combination with CTLA-4 blockade (nivolumab–ipilimumab), PD-1 blockade in combination with LAG-3 blockade (nivolumab–relatlimab), and, for BRAF-mutated melanoma, BRAFi (vemurafenib, dabrafenib, encorafenib) combined with MEKi (cobimetinib, trametinib, binimetinib) in the first or second line treatment [4,5]. Registration studies for ICIs have demonstrated their efficacy in prolonging both overall survival (OS) and progression-free survival (PFS), along with a higher percentage of objective responses (ORR) [[6], [7], [8], [9], [10], [11]]. A notable aspect of these clinical trials is their extensive follow-up periods, which have facilitated the determination of time to subsequent therapies. A case in point is the CheckMate-067 study, which revealed that the median time to subsequent systemic therapy was not reached in the nivolumab-plus-ipilimumab group and was 25.5 months in the nivolumab group and 8.1 months in the ipilimumab group [12]. Subsequent analyses have enabled the assessment of optimal sequencing in cases of *BRAF* mutant patients, as evidenced by the DREAMSeq and SECOMBIT studies; the superiority of ICIs in terms of OS and duration of response (DOR) when used prior to BRAFi/MEKi has been demonstrated [13,14]. However, despite the exceptional outcomes observed with first-line treatment, the disease progresses in approximately 40 % of patients due to acquired treatment resistance [2]. The present use of ICIs in adjuvant treatment also indicates the occurrence of primary resistance. In studies on patients previously treated with PD-1 inhibitors, the combination of immunotherapy with ipilimumab and nivolumab is more effective than ipilimumab alone. The response rate was found to be approximately 30 % (95 % confidence interval [CI]: 18.4––40.6 %), however, it is significantly lower than the 58 % response rate documented in the registration study (95 % CI: 53––64 %) [12,15]. A notable challenge also arises from the substantial adverse events (AEs) of the medications utilized, which, in conjunction with the presence of concomitant diseases or overall health status, result in patient ineligibility for treatment [16]. In the event of progression, particularly among *BRAF* wild-type patients, therapeutic options are limited.

The utilization of stereotactic radiation therapy (SBRT) in the management of oligoprogression, characterized by the progression of a limited number of metastases under active systemic therapy in melanoma, is currently under investigation. This investigation encompasses the effectiveness, safety, and potential for extending the time to the next systemic therapy (TTNST) [[17], [18], [19]]. A growing body of research has demonstrated the efficacy of RT in a variety of clinical settings. Patients diagnosed with oligoprogressive non-small cell lung cancer who underwent RT exhibited significantly improved PFS outcomes compared to those receiving standard of care (hazard ratio [HR]: 0.53, 95 % CI: 0.35–0.81, p = 0.0035) [20]. In renal cancer, this approach resulted in a 1-year OS rate of 92 % (95 % CI: 82–100 %) and a median PFS after RT of 9.3 months (95 % CI: 7.5–15.7) [21]. Few retrospective analyses have confirmed these results in melanoma, with a 1-year PFS after RT of 47.0 % (95 % CI: 35–59) [22]. The incorporation of increased temperature with hyperthermia (HT) has the potential to further enhance these outcomes, as demonstrated in our previous study, achieving 1- and 2-year OS rates of 100 % and 95 % (95 % CI: 90.4–99.9 %), respectively [18]. Less frequently, although recently emphasized, the feasibility of delaying the change of systemic treatment regimen by controlling oligoprogression with RT is assessed, and preliminary results are promising. In a study of metastatic castrate-resistant prostate cancer, the use of SBRT enabled the achievement of a median TTNST of 10.1 months and a median OS of 41.3 months, especially in patients with a limited number of metastases (HR, 0.67; 95 % CI: 0.33–1.36, p = 0.24) [23]. Concurrently, in the context of lung cancer, SBRT has been demonstrated to yield outcomes that are comparable to those achieved through a change of systemic treatment in progressing patients, as evidenced by median OS (32.1 vs. 38.2 months, p = 0.47) and PFS (4.3 vs. 3.4 months, p = 0.6) [24].

In consideration of the aforementioned points, a retrospective analysis was conducted on a consecutive cohort of patients treated with a multimodal therapeutic approach. This approach comprised RT in combination with locoregional HT and systemic treatment, as administered at our institution. The objective of this study was to assess the feasibility of delaying the switch of systemic treatment with RT and RT with HT in patients diagnosed with MM and oligoprogression.

## Methods

### Patients characteristics

Patients with histologically confirmed stage IV melanoma of the skin during systemic treatment, who were additionally treated for oligoprogressive lesions with RT or RT combined with locoregional HT at the Maria Sklodowska Curie National Research Institute of Oncology in Warsaw, Poland, between 2018 and 2023, were included in this study. All patients fulfilled the criteria of oligoprogression, as defined by the ESTRO and EORTC criteria, assuming limited progression of some lesions with stable disease responding to systemic treatment, with a maximum of five progressive metastatic lesions [4,25]. Certain metastatic sites, including the brain or lungs, were excluded due to treatment guidelines, which eliminate these locations from the application of HT. The clinical data were obtained from the medical records and included the age, *BRAF* status, gender, irradiated lesion site, details of the RT, and information about systemic treatment administered during RT or up to a month before/after (Table 1). Response to treatment was assessed by computed tomography (CT) scans and defined according to Response Evaluation Criteria In Solid Tumors 1.1 (RECIST1.1). Follow-up data were obtained in accordance with current guidelines, with CT scans conducted approximately every three months [26,27].

**Table 1:** Characteristics of patients included in the analysis. RT – radiotherapy, *- concomitant systemic treatment administered during RT or up to a month before/after, **-clinical trials based on ICIs.

Characteristics	Number of patients (%)		*p*
	RT	RT + HT	
**All patients**	**74 (47.4)**	**82 (52.6)**	
**All irradiated lesions**	**97**	**87**	
**Number of irradiated lesions per patient**	**1.3**	**1.1**	
**Age**	(years)		0.96
Median	64	62	
Range	31–89	19–90	
**Sex**			0.85
Male	41 (55.4)	47 (57.3)	
Female	33 (44.5)	35 (42.7)	
***BRAF* status**			0.2
*BRAF*(−)	36 (48.5)	44 (53.7)	
*BRAF*(+)	38 (51.4)	38 (46.3)	
Systemic treatment*			0.009
BRAF/MEK inhibitors	12 (16.2)	13 (15.6)	
Nivolumab + Ipilimumab	7 (9.5)	16 (19.5)	
Pembrolizumab	25 (33.8)	22 (26.8)	
Nivolumab	28 (37.8)	18 (21.6)	
Clinical trial**	2 (2.7)	13 (15.9)	
**Treatment line during RT**			
First-line	63 (64.9)	62 (71.3)	
Second-line	25 (25.8)	20 (23)	
Third-line	9 (9.3)	5 (5.7)	
**Metastasis sites number**			
1	31 (41.9)	31 (37.8)	
2–3	38 (51.4)	47 (57.3)	
>3	5 (6.8)	4 (4.9)	
**Target volume** (localization of oligoprogression)			
Lymph nodes	15 (20.3)	39 (47.6)	
Skin and subcutaneous tissue	11 (14.9)	31 (37.8)	
Abdominal cavity (liver, rectum, adrenal glands, kidneys, pancreas, retroperitoneal structures)	48 (64.9)	12 (14.6)	
**Tumor size** (diameter in the two largest dimensions, accessed at the time of RT planning)	**Size (mm)**		**0.81**
Median	46x33.5	45x32	
Range	8x9-163x177	10x10-130x165	

### Radiotherapy and hyperthermia details

**Fig. 1:**
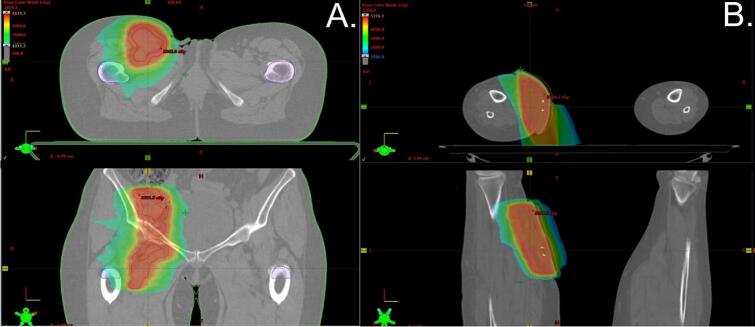
Radiotherapy planning of A- metastatic lesions in the inguinal and internal iliac lymph nodes, B- metastatic lesions in subcutaneous tissue of the right lower limb.

RT planning and delineation were conducted by an experienced team comprising at least two radiation oncologists, with protocols in place for verification and validation (Fig. 1). Fractionation regimens involved delivering mostly 10–50 Gy in 5–10 fractions (Table 2). The biologically effective dose (BED) was calculated assuming an alpha/beta ratio of 2.5 Gy [28]. Immobilization strategies and bolus application were tailored on an individual basis. The gross tumor volume (GTV) was delineated on planning CT in selected patients using image registration with contrast-enhanced magnetic resonance imaging (MRI) or diagnostic contrast-enhanced CT, as required. The decision to define a clinical target volume (CTV) was contingent upon a thorough benefit-risk assessment. The planning target volume was established by expanding the GTV or CTV. Patients were treated using external beam RT using a linear accelerator. Most patients were irradiated with intensity-modulated RT (IMRT).

**Table 2:** Characteristics of employed radiotherapy. RT- radiotherapy, HT- hyperthermia, BED- biologically effective dose.

	**Dose (Gy)**		
**RT total doses4**	**RT**	**RT + HT**	***p***
Median	30	30	<0.001
Range	24–50	10–40	
**RT fraction doses**			0.6
Median	6	6	
Range	5–10	5–10	
**BED for alpha/beta = 2.5 Gy**			<0.001
Mean	120.4	104.7	
Median	102.0	102.0	
**RT fractionation regimens**	Number of lesions		Corresponding BED for alpha/beta 2.5 Gy (Gy)
	**RT**	**RT + HT**	
30 Gy in 5 fractions	29	51	102
40 Gy in 8 fractions	20	2	120
40 Gy in 5 fractions	15	4	168
25 Gy in 5 fractions	12	4	75
30 Gy in 6 fractions	5	9	90
24 Gy in 3 fractions	0	7	100.8
24 Gy in 4 fractions	4	2	81.6
35 Gy in 5 fractions	2	2	133
30 Gy in 3 fractions	0	3	150
50 Gy in 10 fractions	3	0	150
50 Gy in 5 fractions	3	0	250
32 Gy in 4 fractions	2	0	134.4
45 Gy in 5 fractions	2	0	207
40 Gy in 4 fractions	1	0	200
10 Gy in 2 fractions	0	1	30
20 Gy in 4 fractions	0	1	60
**HT**	Number of patients (%)		
Treated with HT	82 (52.7)		
Treated without HT	74 (47.3)		

HT was administered biweekly. For each patient, anthropometric measurements such as body weight and height, along with tumor-specific parameters, were assessed to select the appropriate thermal therapy equipment. The therapeutic objective was to elevate the temperature of the targeted tissue to between 39 °C and 42 °C. After each HT session, an RT fraction was delivered within one hour. Two systems were employed for HT. Firstly, the Celsius TCS system facilitates uniform temperature distribution within the treated area using a water bolus system and an adjustable dual-electrode configuration. This system employs 13.56 MHz electromagnetic waves to generate thermal energy, utilizing capacitive coupling for energy transmission. Secondly, the BSD-2000 3D system is specifically indicated for deep-seated abdominal and pelvic tumors. This equipment encompasses the patient with 24 antennas emitting electromagnetic waves ranging from 75 to 140 MHz and employs specialized algorithms to focus the energy on the target volume. Contraindications for HT comprised implanted medical devices (e.g., pacemakers, stabilizers, or prostheses), significant tumor-related pain unmanageable with pharmacotherapy, severe cardiac or pulmonary conditions, uncontrolled hypertension, recent myocardial infarction or cerebrovascular incident within the past six months, or pregnancy.

### Statistical analysis

The primary endpoint, the duration of clinical benefit compared between RT + HT and RT as sole treatment, was assessed by TTNST, which was defined as the time from RT treatment for oligoprogression until the start of the next line of systemic therapy. Secondary endpoints were PFS and OS; PFS was defined as the time from local treatment to progression. OS was calculated from the start of radiotherapy to death or last follow-up. For patients who were still alive at the time of the analysis, follow-up was censored at the time of the last observation. The overall response to treatment was evaluated in accordance with RECIST 1.1. The Kaplan-Meier method was utilized to estimate the TTNST, PFS, and OS rates, and the median follow-up period was calculated using the reverse Kaplan–Meier estimator. The median follow-up period was 23 months (15.2–34 months). A univariate analysis was conducted using a Cox proportional hazard regression model, and a multivariate analysis was conducted for TNNST. The data on patient characteristics, such as age, gender, irradiated region, and concomitant treatment, have been summarized using descriptive statistics. A comparison of demographic and clinical characteristics between patient groups was performed after analysis of normal distribution using the Shapiro-Wilk test [29]. Continuous variables were compared by Student's *t*-test (for normally distributed values) [30] or the Wilcoxon test (not normally distributed) [31], and categorical variables were compared using Chi-square analysis [32] or Fisher's exact test [33]. The statistical analysis was conducted and presented using R version 4.3.2, with the “survival” package employed for this purpose [34].

## Results

### Characteristics of the patients and radiotherapy details

A total of 156 patients with histologically confirmed stage IV MM who were treated for 184 oligoprogressive lesions with RT combined with locoregional HT were included in this study. The median age of the patients at the time of local treatment was 63 years (19–90 years), and the majority of the included patients were male (56.4 %). 82 patients received RT + HT and 74 had RT as a sole local treatment; all of the patients were treated with systemic therapy during irradiation. The study population included 131 patients (83.9 %) receiving ICIs-based systemic therapy and 25 patients receiving BRAFi/MEKi. 76 (48.7 %) patients had *BRAFV600* mutation. Of these patients, 62 (40 %) had only one metastatic site, and 23 (14.7 %) had brain metastases. The most common metastatic sites were skin and subcutaneous tissue and extra-regional lymph nodes (120/156, 77 %). The data on patient characteristics, such as age, gender, irradiated region, and concomitant treatment, have been summarized in Table 1.

The RT and HT specifics are detailed in Table 2. The most commonly employed fractionation regimens involved delivering 30 Gy in 5 fractions. The calculated mean and median BED were 111.1 and 108.8 Gy, respectively. The majority of patients underwent a 60-minute HT session utilizing the Celsius TCS system, while the remaining patients received 90-minute HT sessions using the BSD-2000 3D system. The general comparison of the basic characteristics of patients from the two groups is displayed in Table 3.

**Table 3:** Comparison of demographic and clinical characteristics of patients included in the analysis. BED- biologically effective dose; ITH- immunotherapy, HT- hyperthermia, Mut- mutant, RT- radiotherapy, Wt- wild-type.

**Variable**	**RT + HT group**	**RT group**	***p***
**Age** median (range)	62 (19–90)	64 (31–89)	0.96
**Sex** F/M	35/47	33/41	0.85
***BRAF* status** Wt/Mut	44/38	36/38	0.27
**Tumor size** median (range)	45 × 32 (10 × 10-130 × 165)	46 × 33.5 (8 × 9-163 × 177)	0.81
**RT total dose** median (range)	30 (10–40)	30 (24–50)	<0.001
**RT fraction dose** median (range)	6 (5–10)	6 (5–10)	0.6
**BED** median (range)	102.0 (30–168)	102.0 (75–250)	<0.001
**Systemic treatment** BRAF/MEKi/ ITH	13/69	12/62	0.009

### Time to the next systemic therapy

**Fig. 2:**
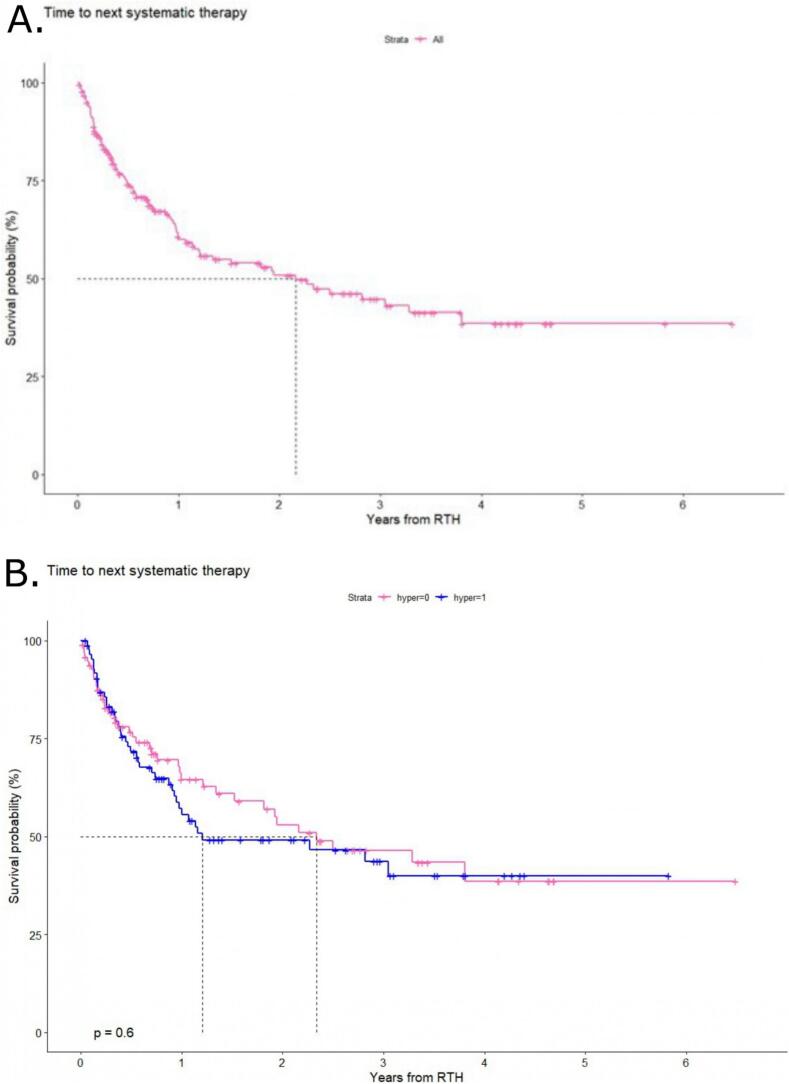
Time to the next systemic therapy (TTNST) for the A.- Overall cohort of patients with melanoma oligoprogression treated with radiotherapy (RT) and RT + hyperthermia (HT). B.- TTNST depending on the treatment used RT vs RT + HT.

**Fig. 3:**
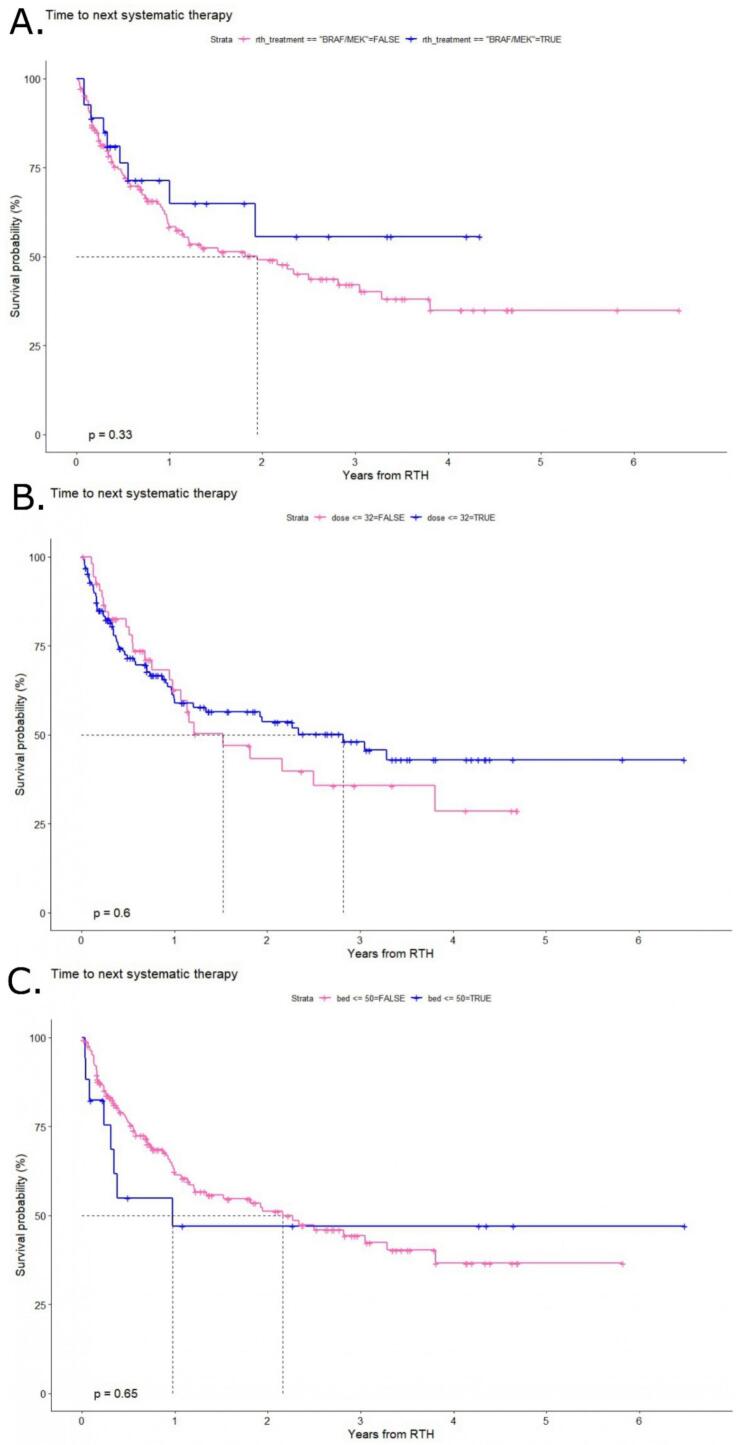
A.- Time to the next systemic therapy (TTNST) for patients with melanoma oligoprogression treated with radiotherapy (RT) and hyperthermia (HT) and RT as a sole method depending on the *BRAF* mutation status. B.- TTNST depending on the median total dose of RT (above/below median). C.- TTNST depending on the median biologically effective dose of RT (above/below median).

The median TTNST (mTTNST) was 26 months (95 % CI: 14.5- NA) for the entire cohort (Fig. 2A). Specifically, the one- and two-year TNST rates were 60.8 % (95 % CI: 53.5–69.2 %) and 50.8 % (95 % CI: 43–60.1 %), respectively. Thus, after both one and two years, the majority of patients did not require a change in systemic treatment. Conversely, the addition of HT to RT in the group of patients undergoing combined treatment resulted in a lower mTTNST of 14 months (95 % CI: 11.4-NA), while in the group with RT as sole treatment, it was 28 months (95 % CI: 18.3-NA), however the difference was not statistically significant (p = 0.6) (Fig. 2B). Subsequent univariate analysis of factors that may have an impact on TTNST revealed no statistically significant differences, as evidenced by the lack of influence of the total RT dose above the median or BED above the median on outcomes (p = 0.6 and 0.65, respectively) (Fig. 3B/C). Furthermore, patients harboring the *BRAFV600* mutation did not demonstrate superior outcomes in comparison to those without the mutation (p = 0.33) (Fig. 3A). This observation was confirmed through multivariate analysis (Table 4), which further substantiated the absence of statistically significant differences in treatment outcomes.

**Table 4:** Multivariate analysis of time to the next systemic therapy factors in metastatic melanoma by the COX proportional hazards regression model. HR- hazard ratio, CI- confidence interval, BED- biologically effective dose, Mut- mutant, Wt- wild-type, ICIs- immune checkpoint inhibitors.

**Factor**	**Class**	**HR**	**95 % CI**	**p-value**
**Hyperthermia**	Yes/no	1.23	0.75–2.0	0.417
**Total dose median**	Above/below	0.80	0.43–1.3	0.397
**Median BED**	Above/below	1.74	0.23–13.0	0.591
**BRAF status**	Mut/Wt	1.22	0.76–2.0	0.408
**Treatment type**	ICIs/BRAFi/MEKi	0.65	0.31–1.4	0.249

### Survival

**Fig. 4:**
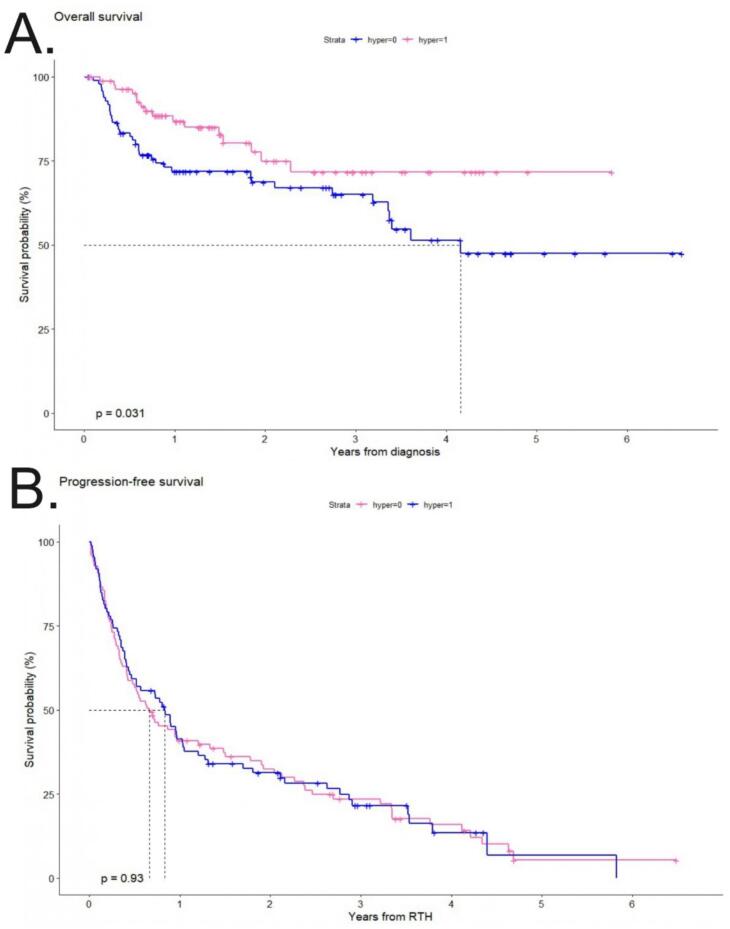
A.- Overall survival for patients with melanoma oligoprogression treated with radiotherapy (RT) and hyperthermia (HT) and RT as a sole method. B.- Progression-free survival for patients with melanoma oligoprogression treated with RT and HT and RT as a sole method.

The median OS from RT was 50 months (95 % CI: 40-NA) for patients who received RT and was not reached at the time of the analysis for patients who received RT + HT, which was significantly better (p = 0.031) (Fig. 4A). The median PFS from local treatment was 8 months (95 % CI: 5.1–16) for patients who received RT and 10 months (95 % CI: 5.6–12.6) for patients who received RT + HT (p = 0.9) (Fig. 4B).

## Discussion

To the best of our knowledge, the present study is one of the first to focus on the possibility of prolonging TTNST in patients with oligoprogression in MM with RT, as well as on the effect of adding HT to RT on TTNS in oncological treatment. In the study, TTNST was effectively prolonged in patients with oligoprogression in MM, regardless of the use of HT. However, its addition was associated with a significant beneficial effect on OS. Consequently, we hypothesize that RT as a sole treatment modality and the combination of RT with HT may delay a change in systemic therapy in patients with MM oligoprogression.

Systemic treatment constitutes the foundation of therapy for MM. ICIs and BRAFi/MEKi have profoundly transformed the landscape of current treatment approaches for these patients, as evidenced by the results of clinical studies demonstrating the significant benefits of systemic treatment [9,12,35]. Consequently, the primary concern is to ensure that patients receive this therapy for a prolonged duration of time. In the MM, current guidelines advocate the inclusion of patients in clinical trials in the second and subsequent lines of treatment. In cases of patients treated with anti-PD-1 monotherapy, dual therapy with two ICIs is also a possible option for BRAF mutant patients after progression on ICIs; the options are BRAFi/MEKi [26]. Another significant option is a rechallenge to previous treatment. Studies have demonstrated that, following progression on PD-1 inhibitors, up to 50 % of patients do not receive subsequent lines of therapy [36]. Rechallenge studies have yielded heterogeneous results, with an emphasis on their positive effect on progressing patients [37]. In the Keynote-006 study, 15 patients received a rechallenge, and 46 % had an objective response to treatment [38]. A retrospective study by the French group revealed an objective response rate (ORR) of 54 % after rechallenge and mPFS of 21 months, with the mOS not yet reached [39]. A substantial analysis revealed an ORR of 40 %, while the mOS was recorded at 9.9 months (95 % CI: 6.8 to 17.9 months), and the median duration of retreatment was 1.6 months (95 % CI: < 1.0–28.3 months) [40]. However, it was demonstrated that the escalation of therapy is more beneficial than returning to monotherapy with a single inhibitor. The mOS was 20.4 months (95 % CI: 12.7–34.8) for combination therapy, compared with 8.8 months (95 % CI: 6.1–11.3) for ipilimumab alone (HR: 0.50, 0.38–0.66, p < 0.0001) [41]. Nevertheless, in the case of ICI's toxicity, significant deterioration of overall performance, and the occurrence of contraindications to therapy, therapeutic options remain highly limited.

The definition of extending systemic therapy can be established using TTNST, which is the time to initiate the subsequent line of treatment. TTNST is one of the less frequently utilized endpoints in studies; however, it has gained clinical value in recent years, particularly in light of the CheckMate-067 study [12]. Furthermore, certain *meta*-analyses suggest that TTNST may be a promising surrogate endpoint for OS in advanced melanoma patients treated with ICIs [42]. The emergence of resistance to systemic treatment may result in the oligoprogression of some metastatic lesions [43]. In clinical practice, significant benefits have been observed from the use of local therapy in these cases, including SBRT and, recently, a combination of radiotherapy with HT. These benefits include the extension of PFS and OS and bear the potential for delaying systemic treatment change [17,18,22]. To date, no study has evaluated the possibility of extending TTNST in melanoma; however, encouraging results have been observed in other cancers. In lung cancer, 36 % of patients undergoing SBRT for oligoprogression also received systemic therapy, of which 58 % achieved a mean break in systemic therapy of 8.5 months (range 1–72 months) [44]. However, the study did not specify the type of systemic therapy used. In ovarian cancer, systemic therapy with RT was used in 22 % of patients, of whom 41 % were able to remain on their previous systemic therapy based on hormone therapy, chemotherapy, or maintenance biological agents after completion of RT [45]. Another study included patients with various diagnoses, including melanoma, to assess the possibility of postponing the change in systemic therapy in patients with primary and acquired resistance to ICIs. The mTTNST outcomes at 24 months (range 7–72 months) were attained in patients with primary resistance, and the median was not reached in the case of acquired resistance. However, this discrepancy was not statistically significant [46]. TTNST has been most extensively studied in prostate cancer, particularly in diverse clinical scenarios. One of the studies, in addition to SBRT as a local treatment, also permitted surgical intervention. However, the included patients had not received systemic treatment, and the time to initiate androgen deprivation therapy was evaluated. The median survival without systemic treatment was 13 months (80 % CI: 12––17 months) for the surveillance group and 21 months (80 % CI: 14–29 months) for the treated group (HR 0.60; 80 % CI: 0.40–0.90) [47]. However, these results were not statistically significant. Another study evaluated 68 patients treated with androgen deprivation therapy after RT, achieving a median time to the next intervention of 15.6 months (95 % CI: 13.8–21.1 months) and observing that 80.9 % of patients remained on the same systemic therapy [48]. The most recent study of prostate cancer patients directly demonstrated that the use of SBRT for oligoprogression during androgen deprivation therapy resulted in prolonged mTTNST. The overall cohort demonstrated an mTTNST of 10.1 months, whereas for patients with one or more untreated metastatic sites at the time of SBRT, TTNST was 8.7 months [23].

In our cohort, the mTTNST was 26 months (95 % CI: 14.5- NA) for the overall cohort, which is slightly more favorable than the results of studies in this field for other diagnoses. Utilizing RT as the sole treatment modality resulted in an mTTNST of 28 months (95 % CI: 18.3- NA). The incorporation of HT was associated with poorer outcomes, with an mTTNST of 14 months (95 % CI: 11.4- NA), which more closely aligns with the findings of other studies. However, it is crucial to acknowledge that these studies encompassed a heterogeneous array of systemic treatment modalities and radiotherapy techniques, thereby complicating direct comparisons. The majority of patients in our study maintained the same treatment regimen for two years, with one- and two-year TTNST rates of 60.8 % (95 % CI: 53.5–69.2 %) and 50.8 % (95 % CI: 43–60.1 %), respectively. The mOS in our cohort was 50 months and not reached, depending on the treatment schedule. The mPFS was 8 months for patients who received RT and 10 months for patients who received RT + HT. This also appears to be a longer period compared to ICI rechallenge and may be particularly important for patients who are not eligible for rechallenge. A notable finding is that the time to next line of treatment in our study was comparable to the CheckMate-067 study, where the median time to subsequent systemic therapy was not reached in the nivolumab-plus-ipilimumab group and was 25.5 months in the nivolumab group and 8.1 months in the ipilimumab group. However, it is important to acknowledge the limitations inherent in our study. Primarily, its retrospective nature, the heterogeneity of RT doses utilized in treatment, and the heterogeneity of the patient population. A significant limitation of the study was selection bias. The patient groups that we analyzed exhibited a heterogeneous distribution of proportions, particularly with regard to the total and fractional doses of RT utilized, which could have directly influenced the results of the analysis. Furthermore, data guiding the optimal application of HT in melanoma remains scarce. Selection of devices, protocols, and energy parameters relied on clinical judgment in the absence of standardized guidelines. Intratumoral temperature was estimated, as invasive monitoring was avoided to preserve safety and feasibility. Celsius TCS was applied using fixed protocols without temperature feedback due to sensor limitations, while BSD-200 provided only indirect measurements. Future research should incorporate advanced modalities such as magnetic resonance thermometry for precise thermal monitoring. Notwithstanding these limitations, we contend that both RT as a sole treatment and RT + HT have the potential to be utilized as a local therapy in oligoprogression MM, thereby enabling a substantial delay in the transition to systemic treatment. This observation assumes particular significance for patients in late treatment lines and those who are not eligible for subsequent lines.

## Conclusions

RT and RT + HT have been demonstrated to extend the duration of systemic treatment in patients, and they appear to be effective options for a specific patient population. These treatments enable the optimization of the choice of subsequent lines of systemic treatment. The combination of RT + HT is suggested to be more efficacious than RT alone in the therapy of oligoprogressive disease during systemic treatment of patients, especially in terms of OS.

## Ethics approval and consent for publication

Each patient, at the beginning of treatment, provided routine informed consent for the use of their treatment and data processing. The study was conducted according to the guidelines of the Declaration of Helsinki. The paper does not report on the use of experimental or new protocols. This study, as part of a project series, was approved by the Local Ethics Committee at Maria Sklodowska-Curie National Research Institute of Oncology (approval number KB/9/2011) to release these data without additional patient consent as patient consent was deemed unnecessary.

## Availability of data and materials

Upon DTA agreement from the PI of the project.

## Funding

Not applicable.

## Declaration of competing interest

The authors declare that they have no known competing financial interests or personal relationships that could have appeared to influence the work reported in this paper.
